# Effect of an interactive E-learning tool for delirium on patient and nursing outcomes in a geriatric hospital setting: findings of a before-after study

**DOI:** 10.1186/s12877-018-0715-5

**Published:** 2018-01-19

**Authors:** Elke Detroyer, Fabienne Dobbels, Andrew Teodorczuk, Mieke Deschodt, Yves Depaifve, Etienne Joosten, Koen Milisen

**Affiliations:** 1 Department of Public Health and Primary Care, Academic Centre for Nursing and Midwifery, KU Leuven, Kapucijnenvoer 35–PB 7001/4, B-3000 Leuven, Belgium; 2 School of Medicine, Griffith University, Gold Coast Campus, Southport, Qld Australia; 3 Campus for Ageing and Vitality, Newcastle upon Tyne, Northumberland Tyne and Wear NHS Foundation Trust, Newcastle upon Tyne, UK; 4 Department of Geriatrics, University Hospitals Leuven, Leuven, Belgium

**Keywords:** E-learning, Delirium, Patient outcomes, Nursing outcomes

## Abstract

**Background:**

Education of healthcare workers is a core element of multicomponent delirium strategies to improve delirium care and, consequently, patient outcomes. However, traditional educational strategies are notoriously difficult to implement. E-learning is hypothesised to be easier and more cost effective, but research evaluating effectiveness of delirium education through e-learning is scarce at present. Aim is to determine the effect of a nursing e-learning tool for delirium on: (1) in-hospital prevalence, duration and severity of delirium or mortality in hospitalized geriatric patients, and (2) geriatric nurses’ knowledge and recognition regarding delirium.

**Methods:**

A before-after study in a sample of patients enrolled pre-intervention (non-intervention cohort (NIC); *n* = 81) and post-intervention (intervention cohort (IC); *n* = 79), and nurses (*n* = 17) of a geriatric ward (university hospital). The intervention included an information session about using the e-learning tool, which consisted of 11 e-modules incorporating development of knowledge and skills in the prevention, detection and management of delirium, and the completion of a delirium e-learning tool during a three-month period. Key patient outcomes included in-hospital prevalence and duration of delirium (Confusion Assessment Method), delirium severity (Delirium Index) and mortality (in-hospital; 12 months post-admission); key nurse outcomes included delirium knowledge (Delirium Knowledge Questionnaire) and recognition (Case vignettes). Logistic regression and linear mixed models were used to analyse patient data; Wilcoxon Signed Rank tests, McNemar’s or paired t-tests for nursing data.

**Results:**

No significant difference was found between the IC and NIC for in-hospital prevalence (21.5% versus 25.9%; *p* = 0.51) and duration of delirium (mean 4.2 ± SD 4.8 days versus 4.9 ± SD 4.8 days; *p* = 0.38). A trend towards a statistically significant lower delirium severity (IC versus NIC: difference estimate − 1.59; *p* = 0.08) was noted for delirious IC patients in a linear mixed model. No effect on patient mortality and on nurses’ delirium knowledge (*p* = 0.43) and recognition (*p* = 1.0) was found.

**Conclusion:**

Our study, the first in its area to investigate effects of delirium e-learning on patient outcomes, demonstrated no benefits on both geriatric patients and nurses. Further research is needed to determine whether delirium e-learning nested within a larger educational approach inclusive of enabling and reinforcing strategies, would be effective.

**Trial registration:**

ISRCTN (82,293,702, 27/06/2017).

## Background

Delirium, defined as an acute and fluctuating disturbance in attention and awareness together with a disturbance in cognition or perception, is the most common hospital complication in older patients [1, 2]. Nurses in particular play a key role in the prevention and early detection of delirium. However, lack of knowledge and competencies required to prevent or manage delirium effectively and negative attitudes towards delirium care, result in adverse patient outcomes, including an increased risk of functional decline, mortality, institutionalisation or dementia [3–7].

Evidence suggests that multicomponent delirium strategies, including educational approaches, improve delirium-related knowledge and recognition of healthcare staff as well as prevent in-hospital delirium [8–11]. Education of nurses and physicians about delirium, with packages including formal presentations or structured courses followed by case-based discussions, feedback, reminders and/or expert local specialist input, are a key element of those multicomponent strategies. Studies have demonstrated the effectiveness of delirium education in improving delirium-related knowledge and recognition skills of nurses and other healthcare staff [10, 11]. Yet, evidence determining its impact on the incidence or in-hospital prevalence of delirium is rather scarce [10, 12, 13].

Moreover, within routine care outside a research environment, these educational initiatives are difficult to implement. Specific challenges include to be time-consuming and labour-intensive to implement and to maintain compliance within systems of care that do not align to good delirium practice [14–16].

E-learning has been identified as an alternative and cost-effective method of delivering education to large groups of hospital staff, and may overcome the challenges of traditional educational approaches [17, 18]. It is proposed that its accessibility, availability, and the use of interactive feedback mechanisms and real care situations make e-learning easier to implement. Arguably, therefore, e-learning at a theoretical level can improve the integration of acquired knowledge into clinical practice, thereby, improving patient outcomes [19, 20].

Two large systematic reviews already evaluated the effect of e-learning education on knowledge, skills and behaviour change in healthcare workers working in the medical (e.g. on management of osteoporosis), psychiatric (e.g. on management of depression), surgical (e.g. on prevention of skin lesion) and nursing (e.g. on prevention of medication errors) field [21, 22]. Though the findings were positive, only one study evaluated the effectiveness of e-learning on patient outcomes [21–24]. Moreover, despite the fact that e-learning gains growing attention in hospital settings and has direct relevance for day-to-day delirium care, no studies exist on the effects of delirium education through e-learning on patient outcomes, and only four studies investigated its effectiveness on nursing outcomes [25–28].

The aim of our study was to explore the effect of a delirium e-learning tool for nurses on in-hospital prevalence, duration and severity of delirium in older patients. The effect on patients’ mortality, and geriatric nurses’ delirium knowledge and their ability to recognize delirium were included as secondary outcomes.

## Methods

### Design, setting and participants

A before-after study (sequential design) was conducted on a geriatric ward of a university hospital in Belgium. The e-learning intervention was implemented over 3 months between 2 periods of data collection i.e. the non-intervention patient cohort (before group, consisting of usual care; enrolled during 4 months) and the intervention patient cohort (after group; enrolled during 4 months). Both cohorts had a follow-up of 12 months from time of admission to the geriatric ward. Dutch speaking patients who were 70 years or older and consecutively admitted to the geriatric ward, were eligible for inclusion. Patients with severe hearing or visual problems, very poor health condition (e.g. palliative patients, patients with unstable cardiac or respiratory problems), isolation because of infectious disease, or those unable to hold a conversation were excluded. Patients who were readmitted during the study period, or had an expected discharge within 24 h after admission were also excluded. Furthermore, all nurses of the geriatric ward were eligible for inclusion. The study was approved by the Medical Ethics Committee of the University Hospitals Leuven, and written informed/proxy consent was obtained in each patient before inclusion.

### Intervention

An on-line self-directed nursing staff educational program on delirium was developed by the research team (ED, FD, EJ, KM). This e-learning tool consists of 11 modules including information about delirium specifics, prevention and treatment strategies for delirium (e.g. including a checklist of 12 risk factors), and information about screening tools for the detection of delirium (with possibility to download the instruments). To help translate new knowledge into practice, the tool incorporates textual information in combination with audio-visual materials, case studies and tests for self-assessment with feedback. The e-learning tool is freely accessible at www.deliriummodule.be. Details about the content, development and feasibility testing of the tool have been described elsewhere [25, 29].

The intervention included (1) a live information session (one hour at the geriatric ward) to offer nurses oral and written information about navigation through the e-learning program, and (2) the completion of six compulsory modules (e.g. ‘occurrence and consequences’, ‘clinical presentation’, ‘exercises in delirium recognition’, ‘predisposing and precipitating risk factors’, ‘screening for delirium, and ‘prevention of delirium’) during a 3-month learning period. The five other modules could be completed on a voluntary basis. The e-learning tool remained available until the end of the study. Participants could access the modules at any time using their personal log-in code. It takes between 5 and 15 min to complete one module. Nurses who did not complete the six compulsory modules within two months were encouraged by the head nurse to complete the course. Additionally, a poster was displayed at the geriatric ward to act as a prompt and further enable knowledge translation.

### Variables and measurements

#### Baseline data

Patient baseline data collected included age, gender, social living circumstances, education level, main diagnosis, number of medications prescribed, number of comorbidities, premorbid functional status, cognitive functioning, confirmed diagnosis of dementia and history of delirium. The number of comorbidities was retained from the modified Charlson Comorbidity Index, and varies between 0 and 13 [30]. The premorbid functional status was evaluated using the Katz Index of activities of daily living (ADL) [31], indicating the level of independence in performing the following six activities scored on a 3-point scale (0 = independent; 1 = partly dependent; 2 = dependent): bathing, dressing, feeding, continence, transfer and toileting. Total score ranges between 0 and 12, with higher scores indicating more dependency. Cognitive functioning was evaluated with the 12-item Mini-Mental State Examination (MMSE) [32]. Total scores vary between 0 and 12, with higher scores indicating better cognitive functioning. Patient baseline data were collected through patient interview, requested from a family member, or based on the medical or nursing records.

Nurse characteristics were collected at the start of the intervention implementation period and included age, gender, work experience as a nurse, percentage employment, day- or night work, highest level of education and delirium education attended in the 5 years prior to the start of the study.

#### Primary outcomes

In-hospital prevalence of delirium was measured with the Confusion Assessment Method (CAM) [33, 34], which was scored after a structured interview including the 12-item Mini-Mental State Examination (MMSE) [32]. Accordingly, delirium was diagnosed when the criteria “(acute onset OR fluctuation), inattention, AND (disorganized thinking OR altered level of consciousness)” were rated as positive on at least one of the measurement points (see procedure).

Duration of delirium was defined as the number of days on which a positive CAM score was obtained.

Severity of delirium was assessed with the 7-item Delirium Index (DI) [35], including inattention, disorganized thinking, altered level of consciousness, disorientation, memory impairment, perceptual disturbance, and disorder of psychomotor activity. Each item was scored on a scale from 0 (absent) to 3 (present and severe) resulting in a total score varying between 0 and 21, with higher scores indicating greater severity.

#### Secondary outcomes

Patients’ in-hospital mortality is defined as the number of deaths occurring while being hospitalized at the geriatric unit. Twelve-month mortality includes all patients that died within 12 months after admission, including cases of in-hospital mortality.

Delirium recognition in nurses was assessed with standardized ‘cases vignettes’ [36], including validated cases about hospitalized patients with dementia, hypoactive delirium, hyperactive delirium, hypoactive delirium superimposed on dementia (DSD) or hyperactive DSD. Before as well as after the e-learning intervention, four slightly different case vignettes were used to avoid recall bias (i.e. dementia, hypoactive delirium, hyperactive delirium and, hyperactive DSD or hypoactive DSD). The behavioral symptoms described in each case had to be scored as dementia, delirium, DSD, normal ageing, depression or none of the options, with each case having only one correct answer. Total delirium recognition (DR) was defined as the number of case vignettes answered correctly (range 0 to 4).

Delirium knowledge in nurses was assessed with the 35-item true-false Delirium Knowledge Questionnaire (DKQ) [25, 37]. Ten items are related to the presentation, symptoms and consequences of delirium, 11 items to the causes and risk factors of delirium, and 14 items to the prevention and management strategies of delirium. The total DKQ score was defined as the number of questions answered correctly and ranged from 0 to 35.

#### Completion of the e-learning tool in nurses

The number of e-learning modules finalized by each nurse was recorded and ranged from 0 to 11.

### Procedure

Patient baseline data, premorbid functional status, number of comorbidities, cognitive functioning, delirium and delirium severity were assessed on the first day after admission to the geriatric ward. In addition, delirium and delirium severity were evaluated on the third, fifth and seventh day after admission to the geriatric ward, and on the day before discharge. From the seventh day after admission, delirium and delirium severity were assessed weekly (e.g. 14th, 21th, day) until hospital discharge. If the patient had delirium on one of the measurement points, the patient was followed up daily until a negative CAM score was obtained. Mortality was recorded during hospitalisation and twelve-month mortality was checked by telephone contact with the patient or his proxy. Procedures for patient assessments in the non-intervention and intervention cohorts were identical. There were no service changes or changes to protocol during the entire study period.

Six study nurses with a master degree performed all assessments. They were trained (i.e. theoretical training of 4 h) by two experts in delirium (ED and KM) according to criteria set in the manuals of MMSE and CAM [33, 34], including evaluation of four clinical cases at the bedside and follow-up discussions. Inter-rater reliability for CAM was κ = 1.00, indicating perfect agreement (inter-rater reliability refers to the agreement of CAM scoring for each study nurse compared with CAM scoring of one of the investigators (ED), and calculated two by two in a random sample of 18 paired observations of enrolled patients).

At the beginning of the one-hour live information session before implementation of the intervention and at the end of the study, nurses received the three questionnaires to assess their baseline data, their knowledge about delirium (DKQ) and their ability to recognize delirium (case vignettes), as described above. Returning a completed questionnaire was considered as informed consent.

### Sample size

According to a power analysis for two cohorts using a two-tailed test of significance with an alpha of 0.10, a beta of 0.30 and an estimated proportion of delirium of 30% for the control cohort [38–40], a sample size of 71 participants was required in each cohort to detect a difference of 50% in prevalence of delirium.

### Blinding

Although patients were blinded to the intervention, nurses and research nurses (data collectors) could not be blinded because of the nature of this study.

### Analysis

Descriptive analysis (i.e. means/median, standard deviations/interquartile ranges, or absolute numbers and percentages) for patients in the control and intervention cohorts, as well as for all included nurses were calculated as appropriate.

A chi square test was used to compare in-hospital prevalence of delirium in the control and intervention cohort. This difference was further explored using a logistic regression model in which a random effect for patient was modelled to account for clustering. Duration of delirium (in days) was compared with the Mann-Whitney U-test. Severity of delirium in the two cohorts was compared using a linear mixed model with a random effect accounting for clustering. The mortality risk was explored with a logistic regression model in all patients and in the subgroup of delirious patients. To correct for baseline differences between both cohorts, baseline functional status score and gender were included in all logistic regression and linear mixed models.

Both in the logistic regression and linear mixed models, a time by group interaction was tested first, and a main effect is estimated in case of a non-significant interaction effect. Non-linear trends of time are considered using quadratic and cubic splines-based trends. The models are likelihood-based and therefore provide valid results in case of a random drop-out pattern, this is when the drop-out chance may be associated with previous observations or covariates in the model [41]. Linear mixed models were performed by using the measurement data on the first, third, fifth, seventh, fourteenth, twenty-first days after admission and those of the day before discharge.

In nurses, delirium recognition scores and delirium knowledge scores before and after introduction of the e-learning intervention were compared using paired t-tests for normally distributed data and the Wilcoxon Signed Rank test for non-normally distributed data. McNemar’s tests were used to test differences in proportions of correct answers on the four ‘case vignettes’.

All tests were two-sided, with *p*-values < 0.05 considered as significant. All analysis were performed on intention-to-treat principle using SPSS, version 21 (SPSS Inc., Chicago, IL) and SAS System for Windows version 9.2 (SAS Institute Inc., Cary, NC, USA).

## Results

### Study participants

**Fig. 1:**
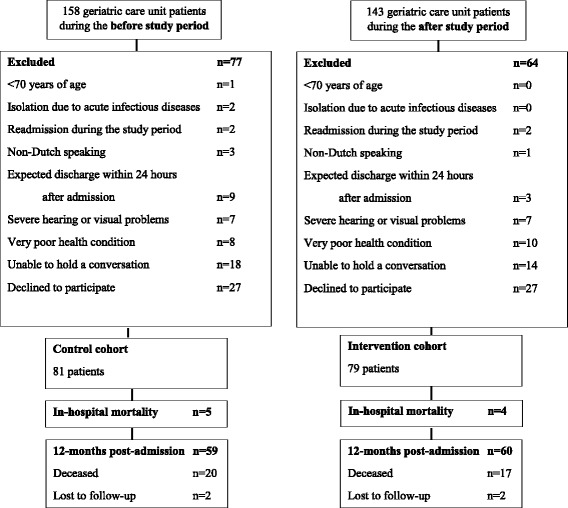
Flowchart

During the before and after study, 153 and 143 patients were consecutively admitted to the geriatric ward, of whom 81 consenting patients were included in the non-intervention and 79 in the intervention cohort (Fig. 1). There were no significant differences in the baseline characteristics of both cohorts, except for gender and premorbid functional status (Table 1).

**Table 1:** Baseline Characteristics of Patients (*n* = 160)

Characteristic	Control cohort (*n* = 81)	Intervention cohort (*n* = 79)	*P*-Value
Age in years, mean (±SD)	83.2 (±5.1)	83.8 (±5.6)	0.486^a^
Female, *n* (%)	34 (42.0%)	51 (64.6)	0.005^b^
Social living circumstances, *n* (%)			0.359^b^
At home, alone	30 (37.1)	32 (40.5)	
At home, with others	31 (38.3)	30 (38.0)	
Nursing home/service flat	18 (22.2)	16 (20.2)	
Other	2 (2.4)	1 (1.3)	
Main diagnosis, *n* (%)			0.531^b^
Heart failure and respiratory insufficiency	10 (12.4)	15 (19.0)	
Infectious disease	25 (30.9)	22 (27.9)	
Gastro-intestinal disease	14 (17.3)	10 (12.7)	
Falls-fractures-osteoporosis	21 (25.9)	15 (19.0)	
Neuropsychiatric disease	5 (6.2)	5 (6.3)	
Cancer	2 (2.5)	6 (7.6)	
Other	4 (4.9)	6 (7.6)	
Number of comorbidities, mean (±SD)	2.7 (±1.5)	2.5 (±1.6)	0.365^c^
Number of medication, mean (±SD)	3.5 (±8.0)	3.2 (±8.0)	0.839^a^
Premorbid Katz ADL score, mean (±SD) (range 0–12)	2.9 (±3.0)	4.4 (±3.5)	0.004^c^
Baseline Mini-Mental State Examination score, mean (±SD) (range 0–12)	8.4 (±3.4)	8.0 (±3.5)	0.509^a^
Dementia, *n* (%)	16 (19.8)	11 (13.9)	0.400^b^
History of delirium, *n* (%)	13 (16.1)	12 (15.4)	1.000^b^

*Abbreviations: SD* standard deviation

^a^Unpaired t-test

^b^Chi-square test

^c^Mann-Whitney U-test

A total of 22 nurses were eligible for inclusion. Five of them dropped-out because of inability to follow the e-learning course during the study period (i.e. no time or long-term sick leave; *n* = 2) or because they were transferred to another unit (*n* = 3). Characteristics of the 17 included nurses are shown in Table 2.

**Table 2:** Characteristics of Nurses (*n* = 17)

Characteristics	
Age in years, mean (±SD)	36.1 (±11.3)
Female, *n* (%)	16 (94.1)
Work experience in years, mean (±SD)	13.3 (±11.1)
Level of education, *n* (%)	
Associate degree in nursing	6 (35.3)
Bachelor degree in nursing	9 (52.9)
Master degree	2 (11.8)
Computer literate, *n* (%)	17 (100)

*Abbreviations: SD* standard deviation

### Completion of the e-learning tool in nurses

Out of the 17 nurses participating, 15 completed the 6 compulsory modules during the implementation period. The remaining 2 completed the 6 modules one month after the implementation period. Moreover, 3 nurses recompleted the 6 compulsory modules plus 2 (*n* = 1) or 5 additional modules (*n* = 2).

### Primary outcomes

#### In-hospital prevalence, duration and severity of delirium

There was no significant difference in the overall proportion of delirious patients in the control (25.9%, *n* = 21) and intervention cohort (21.5%, *n* = 17; *p* = 0.51; Odds Ratio (OR) = 0.47, Confidence Interval (CI) = 0.16–1.42; *p* = 0.18).

The mean duration of delirium was 4.9 (SD 4.8) days in the control and 4.2 (SD 4.8) days in the intervention cohort (*p* = 0.38).

**Fig. 2:**
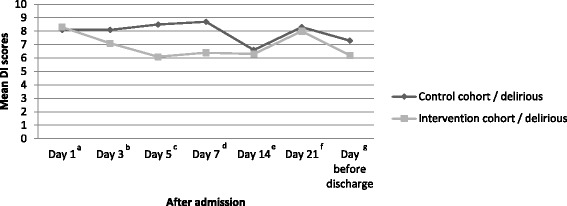
Severity of Delirium. *Abbreviations:* DI = Delirium Index (range 0–21). ^a^ number of delirious patients in intervention/non-intervention cohorts day 1, *n* = 10/*n* = 9. ^b^ number of delirious patients in intervention/non-intervention cohorts day 3, *n* = 6/*n* = 7. ^c^ number of delirious patients in intervention/non-intervention cohorts day 5, *n* = 4/n = 9. ^d^ number of delirious patients in intervention/non-intervention cohorts day 7, n = 6/*n* = 10. ^e^ number of delirious patients in intervention/non-intervention cohorts day 14, *n* = 3/*n* = 4. ^f^ number of delirious patients in intervention/non-intervention cohorts day 21, *n* = 2/*n* = 6. ^g^ number of delirious patients in intervention/non-intervention cohorts day before discharge, n = 1/n = 2

Although the mean DI scores for delirious patients in the intervention cohort were lower than for those in the control cohort on all measurement points, except for day 1 (Fig. 2), linear mixed model analysis noted a trend towards a lower severity score in the intervention cohort (intervention cohort (IC) versus control cohort (CC): Difference Estimate (DE) = − 1.59; 95% CI -3.37 – 0.19; *p* = 0.08).

### Secondary outcomes

#### Patients’ mortality

The mortality risk was calculated for all patients and for delirious patients only. The odds ratios for in-hospital mortality and twelve-month mortality between the non-intervention and intervention cohorts was 0.85 (95% CI 0.20–3.66; *p* = 0.80) and 0.75 (95% CI 0.33–1.71; *p* = 0.50), respectively. For delirious patients, multivariable analysis showed no significant difference in the risk for in-hospital mortality (OR = 3.28; 95% CI 0.40–27.26; *p* = 0.27) and twelve-month mortality (OR = 1.00; 95% CI 0.23–4.37; *p* = 0.99) between both cohorts.

#### Nurses’ delirium recognition (DR)

There were no significant differences in the proportions of nurses who were able to correctly identify dementia (76.5% vs. 94.1%; *p* = 0.37), hyperactive delirium (82.4% vs. 88.8%; *p* = 0.62), hypoactive delirium (52.9% vs. 64.7%; *p* = 1.0) and delirium superimposed on dementia (94.1% vs. 58.8%; *p* = 0.07) before and after the introduction of the e-learning intervention, respectively. No significant improvement in the mean total DR score (3.1 (SD 0.83) vs. 3.1 (SD 0.75), *p* = 1.0, respectively) was noted.

#### Nurses’ delirium knowledge

The mean total DKQ score of nurses before introduction was not significantly different from the score after introduction of the e-learning intervention (29.3 (SD 2.6) vs. 29.9 (SD 3.2); *p* = 0.43, respectively).

## Discussion

To the authors’ knowledge, this is the first study to report effects of delirium education for nurses through e-learning on patient outcomes. Nevertheless, we found no impact of the delirium e-learning tool on the in-hospital prevalence, duration and severity of delirium or mortality in patients, nor on nurses’ knowledge about delirium or on their ability to recognize delirium using case vignettes. Hence, our findings do not support the assumption that e-learning facilitates knowledge acquisition and its integration into clinical practice.

In understanding the findings, important considerations should be taken into account. First, in contrast with previous research [25, 26, 36, 37], our geriatric nurses’ baseline recognition and knowledge levels regarding delirium were already high, likely because of their specific experience with delirious patients and the prevention and management strategies not present in nurses working on non-geriatric wards. As a consequence, one could hypothesise that the effect of e-learning education on nursing and patient outcomes is potentially more favourable when implemented on wards where the clinical experience with delirium is rather limited. Second, the majority of nurses were only exposed to the 6 compulsory modules which exclusively focussed on the prevention and recognition of delirium. Although the state of the science on delirium management is not strong and prevention remains the most important strategy to address delirium [42–44], a lack of completion of all modules available within the tool might in part explain why our e-learning tool failed to affect particularly delirium severity and duration. Third, our findings are in line with a previous study in the broader e-learning literature regarding fall prevention, who did not find an effect of e-learning on patient outcomes either [23]. Overall, studies testing the effectiveness of e-learning in clinical practice is relatively scarce at present, hence, the real value of e-learning has yet to be demonstrated.

Further studies might consider approaches to improve uptake and effect of e-learning. More specifically, educational interventions embedding enabling and reinforcing strategies (guidelines, pocket cards, reminders or feedback) appear to be effective in improving patient outcomes [10, 45]. Therefore, future studies should investigate the efficacy of delirium e-learning integrated within a larger approach of blended-learning education extended with enabling and reinforcing strategies. Moreover, future research should also evaluate the extent to which delirium e-learning can influence behaviour change and positive delirium practice. Examples of clinicians’ behaviour that might optimize patient outcomes are assessing risk factors of delirium, use of screening tools, delirium detection rates, documentation of delirium in notes, or implementation of preventive/management strategies. The fact that most of our nurses did not complete all available e-learning modules indicates that there might be additional factors, such as attitudes and motivation, that could potentially hinder a successful change in clinical practice [46].

Some methodological limitations need to be considered. First, a before/after design was used. More rigorous designs (e.g. cluster randomized trial) might potentially yield different results, although one should realize that education is a social process heavily influenced by contextual factors which cannot be controlled for completely [47]. Second, unlike previous data where post-intervention nursing outcomes were evaluated immediately after exposure to the e-learning education [25–28], we evaluated nurses’ delirium-related knowledge and recognition levels only 4 months after the education implementation period. This four-month interval between the exposure to e-learning education and the measurement of nursing outcomes might have been too long to identify statistically significant improvements in those outcomes. Nevertheless, a clinically significant 12% to 18% higher proportion of correctly identified hypoactive delirium and dementia cases were found, respectively. A lack of statistical significance in those latter nursing findings could be due to the small sample size of nurses. Last, we are aware that we have a high refusal rate in patients. However, it is inherent to this patient population that the drop out is high and the cooperation is limited.

Despite these caveats, this study has several strengths including its prospective design; the repeated assessments during hospitalisation; the use of validated instruments to assess patients’ delirium prevalence and duration, and nurses’ level of recognition; the detailed statistical analysis; the implementation of a well-designed self-directed e-learning tool, and its development via a robust process and feasibility testing.

## Conclusion

Despite the delivery of a well-designed delirium educational e-learning tool, e-learning as an educational approach had neither a direct impact on the in-hospital delirium prevalence, duration and severity or mortality, nor did it improve nurses’ delirium knowledge and their recognition skills. Future studies should therefore focus on evaluating patient outcomes as well as on healthcare workers’ delirium knowledge, behaviour and practices using e-learning within a larger educational approach or quality improvement project with enabling and reinforcing strategies both on geriatric and non-geriatric wards.

## Abbreviations

ADL: Activities of daily living
CAM: Confusion Assessment Method
CC: Control cohort
CI: Confidence interval
DE: Difference estimate
DI: Delirium Index
DKQ: Delirium Knowledge Questionnaire
DR: Delirium recognition
DSD: Delirium superimposed on dementia
IC: Intervention cohort
MMSE: Mini-Mental State Examination
OR: Odds ratio
SD: Standard deviation
